# Responses of the Soil Microbial Community to Salinity Stress in Maize Fields

**DOI:** 10.3390/biology10111114

**Published:** 2021-10-29

**Authors:** Yaling Hou, Wenzhi Zeng, Menglu Hou, Zhao Wang, Ying Luo, Guoqing Lei, Bo Zhou, Jiesheng Huang

**Affiliations:** 1 State Key Laboratory of Water Resources and Hydropower Engineering Science, Wuhan University, Wuhan 430072, China; hyl_cristinehou@whu.edu.cn (Y.H.); luoying1997@whu.edu.cn (Y.L.); leiguoqing1001@whu.edu.cn (G.L.); 00011579@whu.edu.cn (J.H.); 2 State Key Laboratory of Hybrid Rice, The Institute for Advanced Studies, Wuhan University, Wuhan 430072, China; houmenglu@whu.edu.cn (M.H.); wangz201530@126.com (Z.W.); 3 College of Water Resources and Civil Engineering, China Agricultural University, Beijing 100083, China; zhoubo89@cau.edu.cn

**Keywords:** soil salinization, bacteria, fungi, soil microbial diversity, soil microbial community composition

## Abstract

**Simple Summary:**

Soil microorganisms are the core of maintaining soil ecological functions. Recognition of microbial community diversity in saline soil contributes to nutrient management and crop production. Meanwhile, microbial activity is easily affected by changes in soil properties. This study addressed how the composition and diversity of bacterial and fungal communities changed under different saline conditions to identify the sensitivity of bacteria or fungi to salinity. The primary objective was to evaluate the relationship between soil’s microbial community diversity and soil’s physicochemical factors, and to explore the vital microbial predictors in salinized soil. The results showed that Firmicutes and Bacteroidetes are pivotal in salinized soil, and this finding can provide guidance for the demand for plant rhizosphere growth-promoting bacteria as a bioindicator in saline soil.

**Abstract:**

To investigate the diversity and structure of soil bacterial and fungal communities in saline soils, soil samples with three increasing salinity levels (S1, S2 and S3) were collected from a maize field in Yanqi, Xinjiang Province, China. The results showed that the K^+^, Na^+^, Ca^2+^ and Mg^2+^ values in the bulk soil were higher than those in the rhizosphere soil, with significant differences in S2 and S3 (*p* < 0.05). The enzyme activities of alkaline phosphatase (ALP), invertase, urease and catalase (CAT) were lower in the bulk soil than those in the rhizosphere. Principal coordinate analysis (PCoA) demonstrated that the soil microbial community structure exhibited significant differences between different salinized soils (*p* < 0.001). Data implied that the fungi were more susceptible to salinity stress than the bacteria based on the Shannon and Chao1 indexes. Mantel tests identified Ca^2+^, available phosphorus (AP), saturated electrical conductivity (EC_e_) and available kalium (AK) as the dominant environmental factors correlated with bacterial community structures (*p* < 0.001); and AP, urease, Ca^2+^ and EC_e_ as the dominant factors correlated with fungal community structures (*p* < 0.001). The relative abundances of Firmicutes and Bacteroidetes showed positive correlations with the salinity gradient. Our findings regarding the bacteria having positive correlations with the level of salinization might be a useful biological indicator of microorganisms in saline soils.

## 1. Introduction

Soil salinization is a major concern of the international community, which poses a considerable threat to ecosystem health [1]. Taking China as an example, the northwest region accounts for 71% of total area, of which 70% is saline-alkali land. Meanwhile, the largest province of China is Xinjiang, and saline-alkali land accounts for more than 32.6% [2,3]. Saline soil has long been known to be an extremely harsh habitat for life; even so, some active microbial communities still exist in it [4]. It is well known that soil microbes are of pivotal importance in integral natural ecosystems, such that the abundance and activity of saline microorganisms determine the agricultural land sustainable productivity. The activity of microorganisms is directly affected by environmental changes [5]. Thus, understanding information on microbial diversity and distribution is indispensable to comprehend microbial processes in extreme agricultural ecosystems.

The microbial community is an important driving factor of soil’s biogeochemical cycle; moreover, microbial activity is easily affected by changes in soil structure and properties [6,7]. However, it is unclear whether bacteria or fungi in soil are more sensitive to increased soil salinization. Chowdhury et al. [8] reported that fungi are more sensitive to salt than bacteria, but higher sensitivity to salinity stress in bacteria has also been reported [9,10,11]. Rath et al. [12] claimed that the growth of bacteria was significantly inhibited by salinity, and yet fungal growth was unrelated to salinity, indicating a higher fungal tolerance to salinity. In addition, the dominant factor affecting bacterial and fungal community structure in saline soils is still a topic of active debate. For example, for bacteria, Hollister et al. [13] showed that the overall soil bacterial communities shared significant correlations with the soil water content, phosphorus and soil organic carbon content, rather than salinity. In contrast, salinity under halophytic vegetation was the main determinant of bacterial communities in saline-alkali land [4,12,14]. With respect to fungi, soil salinity is a decisive factor in the mangroves’ fungal community [15,16]. However, Rath et al. [12] documented that fungi respond to the indirect effects of salinity related to reduced plant C inputs. Therefore, we mainly studied the salt sensitivity of different types of saline bacteria and fungi, and attempted to determine the main environmental factors that affect the community structures.

The microbial growth and activity were negatively affected by soil salinity, such as salt toxicity and decreased water availability, leading to limited energy substrate availability for microorganisms [17,18]. Nevertheless, soil microorganisms are capable of adapting to or tolerating the osmotic stress induced by soil salinity, especially when frequently faced with such salinity stress conditions [9]. Some microorganisms also have the ability to thrive in ponds of high salinity [19], which indicates the evolutionary potential in microbes. In addition, microbial communities with high tolerance and resistance can be enriched in stressful environments. The salt-tolerant rhizospheric beneficial microorganisms are expected to be applied and promoted in the future for saline-alkali soil bioremediation.

In this study, sequencing technology was applied to investigate the diversities of soil bacterial and fungal communities in soils with three different salinities. The purposes of this research were (i) to determine how the composition and diversity of bacterial and fungal communities changed under different saline conditions, (ii) to identify the sensitivity of bacteria or fungi to salinity, (iii) to evaluate the relationships between soil microbial community diversity and soil physicochemical factors and (iv) to explore the vital microbial predictors in salinized soil.

## 2. Materials and Methods

### 2.1. Sampling and Soil Physicochemical Characteristics

Samples, including bulk and rhizosphere soil, were collected at three types (S1:0.36 ds/m; S2:3.69 ds/m; S3:6.72 ds/m) of saline soil samples in the October 2019 at Yanqi, Xinjiang Province, China (41°91′ N, 86°49′ E, elevation 1061 m). At each sampling area, apparently healthy maize plants were collected. Three replicate samples were collected at each salinity soil. Plants were uprooted with forked spades. We carefully shook off the loosely attached soil as bulk soil and brushed off the tightly attached soil to collect rhizosphere soil from each plant (total 18 soil samples). Each bulk soil (S1_BS, S2_BS, S2_BS) and rhizosphere soil sample (S1_RS, S2_RS, S2_RS) was then passed through a 2 mm mesh to remove plant roots and other plant material. During the sampling process, sterile paper was used to wipe the residue attached to the spade and disinfect before the next soil sample was collected to avoid contamination between treatments and to keep the sample fresh. At the same time as collecting the maize, bulk soil samples were collected in depth of 10–15 cm. Finally, 50 mL sterile Falcon tubes were used to store soil samples and then transferred to the laboratory [20]. All rhizosphere and bulk soil were liquid nitrogen frozen after collection.

All rhizosphere soil and bulk soil samples were split into two sub-samples in the ice. One subsample at room temperature was air dried and then sieved through a 2 mm sieve for detecting the soil properties, and the other remaining 20 g soil samples were collected and stored at −80 °C for microbial community sequencing. The soil physical and chemical parameters involving the soil electrical conductivity (EC_e_), pH, soil particle composition (including clay, silt, sand proportion), soil ion (including K^+^, Na^+^, Ca^2+^, Mg^2+^) values, soil organic matter (SOC) level, soil nutrition (including available nitrogen (AN), available phosphorus (AP), available potassium (AK)) and soil enzyme activity (including alkaline phosphatase (ALP), invertase, catalase (CAT) and urease) were measured using standard soil testing procedures [21,22,23]. The basic soil physical and chemical properties are shown in Table 1.

### 2.2. Soil DNA Extraction and Sequencing

The Power Soil DNA Isolation Kit (Omega Bio-tek, Norcross, GA, USA) was used to extract fresh soil DNA following the manufacturer’s instructions. Qubit DNA probes (Invitrogen, from Thermo Fisher Scientific, Waltham, MA, USA) were used to quantify nucleic acids, and quality was evaluated by spectrophotometry (A260/A280 ratio). In brief, to explore the bacterial and fungal communities, amplicon libraries were constructed using eubacterial primers: the V3-V4 regions of the bacterial 16S rRNA gene were amplified by 338F (5′-ACTCCTACGGGAGGCAGCAG-3′) and 806R (5′-GGACTACHVGGGTWTCTAAT-3′) primers [24,25], and the fungal internally transcribed spacer (ITS) gene was amplified using ITS1F (5′-CTTGGTCATTTAGAGGAAGTAA-3′) and ITS2R (5′-GCTGCGTTCTTCATCGATGC-3′) primers [26]. The Supplementary Materials provide sequencing data process.

### 2.3. Statistical Analyses

The alpha diversity (i.e., Shannon diversity, Chao1 richness and coverage) was determined using the “vegan” package in R (version 3.6.2) [27]. Soil basic indexes (ECe, pH, clay, silt, sand, K^+^, Na^+^, Ca^2+^, Mg^2+^, SOC, AN, AP, AK, ALP, invertase, urease and CAT) were evaluated using one-way ANOVA. The differences between the means were determined using the Duncan test at a probability level of *p <* 0.05 in SPSS (version 25.0; IBM, Armonk, NY, USA). Principal coordinate analysis (PCoA) based on Bray−Curtis similarity distances were carried out in the R package vegan [28]. Mantel tests with 999 permutations were used to examine the correlation between environmental distance and microbial community distance within the R vegan package [29]. The relationships between the soil dominant environmental factors (Ca^2+^, AP, EC_e_, AK and urease) and the soil microbial community were analyzed by using redundancy analysis (RDA). The correlations between the basic soil physical and chemical properties and the specific bacterial and fungal phyla relative abundances were analyzed by using Spearman’s analyses in the ggcorrplot package in R (version 3.6.2) [30].

## 3. Results

### 3.1. Soil Physicochemical Properties

Table 1 summarizes the diversity of soil physicochemical properties at three soil salinization levels. The bulk soil salinity varied significantly, ranging from 0.36 to 6.72 ds/m. Soils from the three salinization levels had significant differences in EC_e_ (*p* < 0.05). In terms of cation content, nutrients and soil enzyme activity, the soil samples also varied from each other (Table 1). In contrast, the soil cation values were significantly elevated in S3, and the cation content in the rhizosphere soil was lower than that in the bulk soil (*p* < 0.05). The soil nutrient content and enzyme activity decreased with soil salinity increasing, as indicated by SOC, AN, AP, AK, ALP, invertase, urease and CAT (*p* < 0.05), and basically, there was no significant difference between the rhizosphere and the bulk soil for the same salinity of soil. In addition, there was no statistically significant difference in the soil particle composition for the three salinization levels (*p* > 0.05).

### 3.2. Soil Microbial Distribution and Diversity

A total of 557,735 high-quality V3-V4 16S rRNA Illumina sequences and 1,274,864 high-quality ITS sequences from the soil samples at the three salinization levels were generated. Based on OTUs (operational taxonomic units) at 3% dissimilarity, the sequences of all the samples were clustered into 5609 bacterial OTUs and 1110 fungal OTUs. Rarefaction curves showed that within the range of 5000 sequencing reads, all soil sample curves increased sharply, and then the curves had a tendency to reach a saturated plateau, indicating that the generated data were sufficient for further analysis (Figure S1). Rank-abundance curves revealed that most of the sequences belonging to rare microorganisms had only several sequence tags, yet highly abundant bacteria and fungi in the soil samples were relatively rare (Figure S2).

**Figure 1:**
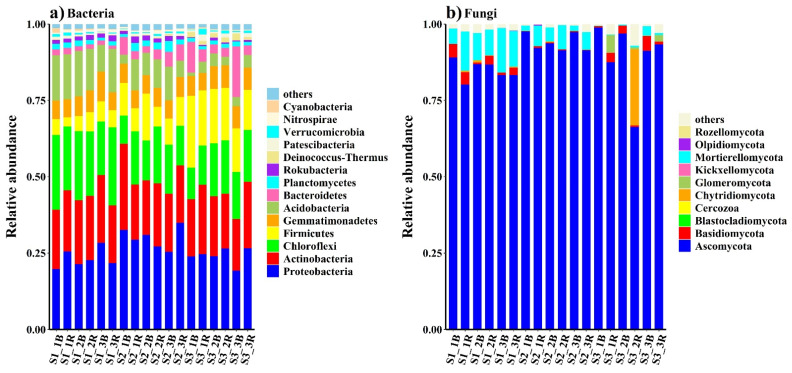
The relative abundances of the bacterial (**a**) and fungal (**b**) phyla in soils with different salinity levels. Less than 1% abundances were classed as “others.”

The bacterial sequences were from 36 phyla, 100 classes, 274 orders, 498 families and 973 genera. Most of the sequences (90%) were classified as bacterial phyla. The predominant phyla (relative abundance >5%) in all the soils were Proteobacteria (26.2%), Actinobacteria (20.3%), Acidobacteria (8.41%), Chloroflexi (17.3%), Firmicutes (10.6%) and Gemmatimonadetes (6.62%), which together accounted for more than 89.4% of the bacterial sequences (Figure 1a). In addition, Bacteroidetes (3.36%), Planctomycetes (1.67%) and Rokubacteria (1.18%) were present at low relative abundances, and another 17 rarer phyla were also identified (Table S1). The fungal sequences were from 10 phyla, 31 classes, 73 orders, 145 families and 254 genera, and the predominant phyla were Ascomycota (89.3%) and Mortierellomycota (5.29%), which accounted for more than 94.5% of the fungal sequences (Figure 1b). In addition to Basidiomycota (1.63%), the relative abundance of Chytridiomycota (1.48%) was low, and six other scarce phyla were identified (Table S2).

**Figure 2:**
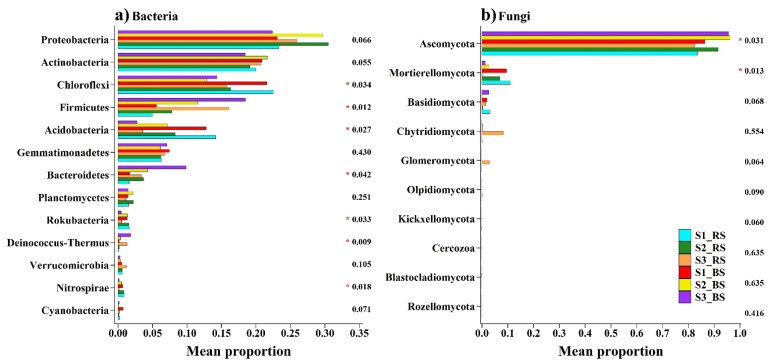
The relative abundances of phylum bacteria (**a**) and fungi (**b**) were significantly different between the samples with different salinization levels. A Kruskal–Wallis H test was used to assess the significance of the differences among the indicated treatments. Note: * *p* < 0.05.

Figure 2 demonstrates the differences in the predominant phyla of bacteria and fungi at the three salinization levels. Comparing S1, S2 and S3, the relative abundances of Chloroflexi, Acidobacteria, Rokubacteria and Nitrospirae were significantly (*p* < 0.05) lower in the high-salt S3 soil compared to S1 and S2. Conversely, the relative abundances of Firmicutes, Bacteroidetes and Deinococcus-Thermus were both significantly (*p* < 0.05) higher in the S3 high-salt soil (Figure 2a). With respect to fungi, only Mortierellomycota (*p* < 0.05) had a greater relative abundance in S3 high-salt soil (Figure 2b).

**Figure 3:**
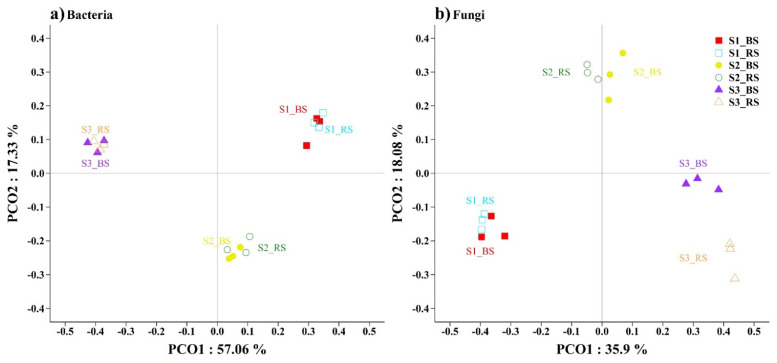
The bacterial community (**a**) and fungal community (**b**) structures were evaluated using PCoA plots of the Bray–Curtis distances.

PCoA served to demonstrate the bacterial and fungal soil samples’ clustering results based on the Bray–Curtis distance. The soil samples from S1 (including S1_BS and S1_RS), S2 (including S2_BS and S2_RS) and S3 (including S3_BS and S3_RS) were distributed in different quadrants, indicating that these soil samples had substantial environmental heterogeneity. Nevertheless, the bulk soil and the rhizosphere soil at the same sampling point were in the same quadrant (Figure 3). These results reveal significant separation of the S1, S2 and S3 communities (ANOSIM, *p* < 0.05, Table S3).

### 3.3. Soil Microbial Communities Associated with Environmental Factors

Bacterial and fungal alpha-diversity indexes are shown in Table S4. The microbial diversity varied greatly with Shannon index (which ranged from 5.99 to 6.52 for bacteria and from 2.51 to 3.89 for fungi) and the Chao1 index (which ranged from 2641 to 3099 for bacteria and from 136 to 456 for fungi) across the soils (Table S4). The study showed that for both the bacteria and the fungi, the alpha-diversity Shannon index was negatively influenced by the soil salinization (*p* < 0.05) and positively influenced by soil nutrition and enzyme activities (*p* < 0.05). Nevertheless, for Chao1 and OTU richness, the bacteria and fungi had opposite relationships with each variable; the bacteria were positively influenced by the soil salinization (*p* < 0.05) and negatively affected by soil nutrition and enzyme activities (*p* < 0.05). The fungi were negatively influenced by soil nutrition and enzyme activities (Table 2).

**Figure 4:**
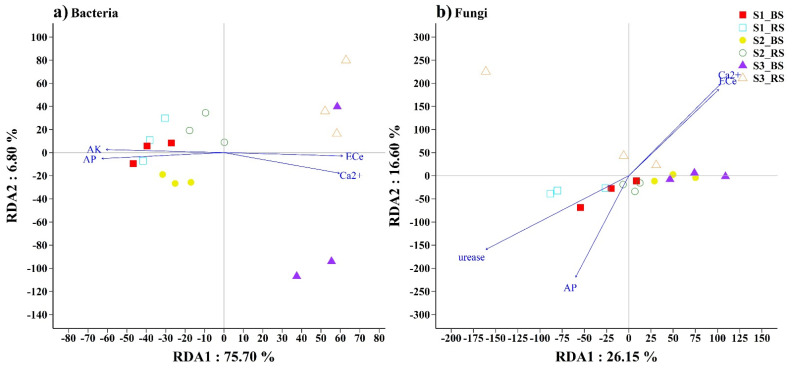
The relationships between dominant soil physicochemical properties and bacterial (**a**) and fungal (**b**) community structures were assessed using redundancy analysis (RDA).

The Mantel tests identified Ca^2+^, AP, EC_e_ and AK as the dominant environmental factors correlated with the bacterial community structures and AP, urease, Ca^2+^ and EC_e_ as the dominant factors correlated with the fungal community structures (Table S5). The significant correlations were confirmed by the linear regression relationships between the dominant environmental factor and the Shannon index (*p* < 0.05), as shown in Figure S3. In the RDA bioplots, the combination of RDA1 and RDA2 variables explained 82.5% of the bacterial community’s variation and 42.75% of the fungal community’s variation (Figure 4).

**Figure 5:**
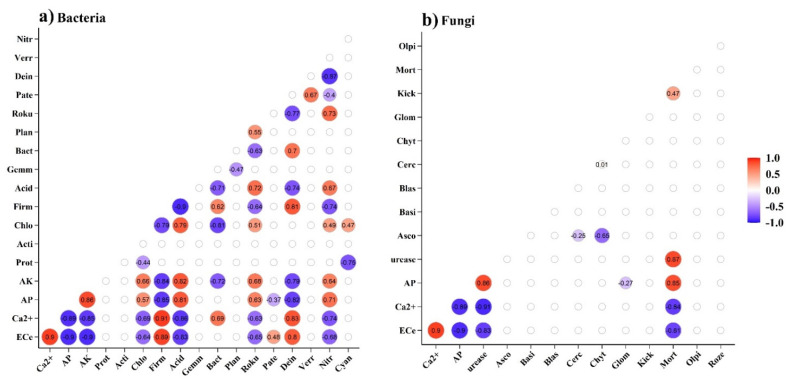
Spearman’s correlation analysis of the relative abundance of the soil’s bacterial community (**a**) and fungal community (**b**) with dominant soil property parameters. The color depth indicates the size of the correlation value. Note: Prot, Proteobacteria; Acit, Actinobacteria; Chlo, Chloroflexi; Firm, Firmicutes; Acid, Acidobacteria; Gemm, Gemmatimonadetes; Bact, Bacteroidetes; Plan, Planctomycetes; Roku, Rokubacteria; Pate, Patescibacteria; Dein, Deinococcus-Thermus; Verr, Verrucomicrobia; Nitr, Nitrospirae; Cyan, Cyanobacteria; Asco, Ascomycota; Basi, Basidiomycota; Blas, Blastocladiomycota; Cerc, Cercozoa; Chyt, Chytridiomycota; Glom, Glomeromycota; Kick, Kickxellomycota; Mort, Mortierellomycota; Olpi, Olpidiomycota; Roze, Rozellomycota.

Spearman’s correlation analysis results indicated that the bacterial abundances of Firmicutes, Deinococcus-Thermus and Bacteroidetes were positively related with the soil Ca^2+^ and EC_e_ values (*p* < 0.05) and inversely correlated with the AK and AP values. Additionally, Acidobacteria, Nitrospirae, Chloroflexi and Rokubacteria had positive correlations with the soil AK and AP values, which inversely correlated with the soil Ca^2+^ and EC_e_ values (Figure 5a). With respect to fungi, Mortierellomycota was significantly positively correlated with the AP and urease values and negatively correlated with the Ca^2+^ and EC_e_ values (Figure 5b).

## 4. Discussion

### 4.1. Responses of Soil Properties to Salinity

At the study site, soil salinity stress is the main cause of crop growth restriction [31]. Typically, Zhao et al. [4] indicated that the relationship between pH value and EC in saline-alkali soil exhibits a significant correlation. Here, the soil EC_e_, pH, K^+^, Na^+^, Ca^2+^ and Mg^2+^ increased with the increase of salinization degree. In general, the K^+^, Na^+^, Ca^2+^ and Mg^2+^ values in the bulk soil were higher than those in the rhizosphere soil, with significant differences in S2 and S3 (*p* < 0.05). The lower content of salt ions in the rhizosphere soil could be explained by root exudates, which were mixtures of many small-molecule compounds, including fatty acids, amino acids, organic acids, sugars and secondary metabolites [32]. Root exudates could reduce salt stress. Studies have shown that the roots of peanut, maize and barley have strong changes in rhizosphere chemistry and biochemistry, which especially affect salt ions’ (Na^+^ and Cl^−^) availability [33].

The values of SOC, soil nutrients (AN, AP, AK) and enzyme activities (ALP, invertase, urease, CAT) in bulk soil and rhizosphere soil decreased as the salinity increased. Abiotic stresses, such as salinity, could suppress the enzyme activity and nutrient concentrations [34]. This might be because a high salt concentration and the toxicity of certain ions lead to nutrient imbalances in microbial growth and enzyme synthesis [35]. In addition, the enzyme activity of bulk soil was lower than that of rhizosphere soil in one study, which was the result of microbial activity caused by root exudates and enzyme release [36]. This result was confirmed in the present study.

### 4.2. Responses of the Bacterial and Fungal Communities to Salinity

To study the microbial community’s composition along the salt gradient, the bacterial community structures and fungal community structures were analyzed at the phylum level. Of all the saline soil samples, bacterial and fungal relative abundances at the phylum level were different. The bacteria were mainly made up of Proteobacteria, Actinobacteria, Chloroflexi, Firmicutes, Acidobacteria and Bacteroidetes, while the fungi were mainly composed of Ascomycota and Mortierellomycota. Furthermore, the results showed that the Firmicutes’ and Bacteroidetes’ relative abundances were positively related with the saline gradient; i.e., the relative abundances increased as the salinity increased.

Microbial communities can respond rapidly to the environmental changes caused by changes in soil salinity. In the current study, the diversity and evenness of bacteria and fungi decreased along a saline gradient (Table S4). Soil salinity caused significant changes in the soil’s microbial community structure, and Herlemann et al. [37] also reported the same results in estuarine and marine environments. One possible explanation is that salt accumulation in the soil increased the extracellular osmotic pressure [38], and numerous microorganisms failing to acclimatize to osmotic stress might have died or become inactive, thereby decreasing microbial diversity and evenness. The present study held that the richness of the rhizosphere soil was lower than that of the bulk soil, which agrees with the results of Thompson et al. [39]. In addition, the richness of bacteria was higher than that of fungi under the same salinity, and the bacterial richness increased along the salt gradient (Table S4). This result indicates that there were some salt-tolerant or halophilic bacteria in the high salinity soil, so as to improve the richness of bacteria. Meanwhile, the bacteria that survived in salty environments had adapted to grow optimally in the high salinity environment to maintain the balance of the intracellular environment [40]. Many fungi were not adapted for such conditions, the reason being that the growth of fungal mycelium was inhibited as the NaCl concentration increased. Soil salinity could directly inhibit fungal mycelial growth through ionic toxicity or indirectly increasing osmotic stress via ion concentrations in the soil [41]. PCoA demonstrated that the soil’s microbial community’s structure significantly changed with different levels of soil salinization. Concurrently, the fungi were less tolerant to salt than the bacteria, based on the Shannon and Chao1 indexes. Consequently, we propose that in the screening of some salt-tolerant plant growth-promoting microorganisms (PGPMs), bacteria should be emphasized.

### 4.3. Responses of Microbial Distribution to Physicochemical Properties in Saline Soil

An important objective of microbial ecology research is to understand the relationships between environmental variables and soil microbial community structure [42]. Salinity’s contributions to microbial communities in soils have been a debated matter over the past few years because of evidence to the contrary. For example, in the majority of studies, saline soil adversely affected the microbial community and its activities in natural saline soil [43,44,45]. This was probably because increasing salt concentration and specific ion toxicity caused a nutritional imbalance for microbial growth and enzyme synthesis [35]. Conflicting results of the salinity’s effects on soil microbial activities, however, have also been reported. Marinari et al. [35] found that soil salinity and sodicity increased both biochemical activity and the functional diversity of microbial communities. This result was consistent with present result. In the present study, Ca^2+^, AP, EC_e_ and AK values were the dominant environmental factors related to the bacterial community structure; and the values of AP, urease, Ca^2+^ and EC_e_ were the dominant factors correlated with the fungal community structure (Table S5), which in turn altered the microbial functional communities. This result indicates that different environmental factors had different effects on microbial diversity. Consistently, Pei et al. [46] showed environmental factors that affected the diversity, composition and structure of microbial communities. Most salts are known to dissolve easily in water, resulting in a solution containing various ions. Therefore, pore water in saline soil contains varieties of dissolved ions, such as Na^+^, Ca^2+^, NH_4_^+^, Cl^−^ and SO_4_^2−^ [38]. Ca^2+^ is associated with a wide range of bacterial functions, including pathogenicity, differentiation, chemotaxis, cell cycle and heat shock [47]. Xia et al. [48] and Xue et al. [49] indicated that Ca^2+^ and EC_e_ are closely correlated with the bacterial community’s structure. According to this study, the critical roles of Ca^2+^ and EC_e_ in shaping the community structure of bacteria and fungi were well characterized, denoting that the Ca^2+^ and EC_e_ contents act as decisive factors in affecting populations and activity of soil bacteria in Xinjiang saline soils. In addition, Rietz and Haynes [50] implied that soil nutrient concentrations and soil enzyme activity were sensitive indicators of the soil ecosystems stress and severely hampered in salinized soil. Interestingly, soil bacterial community composition was significantly affected by available potassium and phosphorus in wild saline soils and tropical ecosystems [51]. In combination with other soil factors, available potassium and phosphorus influence the individual life cycles of bacteria and fungi through competitive strategies, thereby affecting the community structure of bacteria and fungi [2]. For example, the salinized effect on soil microbial communities might vary depending on some specific salt ions. Therefore, Ca^2+^ ions might indirectly affect microbial communities.

Additionally, Spearman’s correlation analysis revealed highly covariable relationships between the core bacterial variables and significant environmental variables. Our results showed that predictors varied with the soil types in Xinjiang, China. Acidobacteria, Nitrospirae, Chloroflexi, Rokubacteria and Mortierellomycota were vital predictors of nutrients in biocrusts, likely owing to their diverse metabolism. Boone et al. [52] reported some *Nitrospirae* genera were aerobic chemolithotrophs. In our study, Nitrospirae showed the excellent relative abundance in the low salt soil S1, with slightly elevated contents of nutrients and enzymes, which could participate in the nutrient cycle. Chloroflexi could provide energy through photosynthesis, the degradation of plant-derived compounds and the decomposition of organic matter [53].

Firmicutes and Bacteroidetes were important and unique predictors of soil salinization. Hashmi et al. [54] also reported that Firmicutes were associated with the development of soil bioremediation for sustainable agriculture. Among the phyla Firmicutes, genus *Bacillus* members were probably widely used beneficial microorganisms in agroecology. *Bacillus*-like organisms predominated in salinized soil environments because of their ability to form spores and Gram-positive cell walls [55]. Strains of *Bacillus* halophilus have numerous advantages, such as promoting plant growth, producing industrially important enzymes and participating in the bioremediation of toxic chemicals [55]. At the same time, a halotolerant *Oceanobacillus* sp. with 1-aminocyclopropane-1-carboxylate (ACC) deaminase activity was used to inoculate wheat containing a NaCl concentration of up to 200 mM, which caused wheat growth to increased [54]. In addition, the genus *Planococcus rifietoensis* SAL-15 of Firmicute phylum, which was separated from wheat rhizospheric salinized soil, exhibited some characteristics of secreting indoleacetic acid (IAA), phosphorous solubilization and ACC deaminase activity. Application of *P. rifietoensis* SAL-15 could improve wheat growth and yield under salt stress, alleviating the deleterious effect of salinity [56]. Bacteroidetes tended to be a dominant phylum within the soil microbiota, reminiscent of human and animal intestines [57]. *Bacteroidetes* species could secrete a variety of carbohydrate active enzymes (CAZymes) which target a variety of glycans in the soil, thereby having the ability to proliferate. *Bacteroidetes* species in the environment are thought to specialize in the biosphere, in complex organic matter degradation, especially in the form of polysaccharides [58]. A study involving soils from 18 different sites showed that Bacteroidetes were correlated with higher electrical conductivity [59]. In fact, Bacteroidetes are abundant in mesotrophic and eutrophic waters and are usually associated with high nutrient values [59,60]. Taken together, these results indicate that Firmicutes and Bacteroidetes were pivotal in salinized soil in Xinjiang, China, which could provide guidance for identifying rhizosphere growth-promoting bacteria (PGPB) as bioindicators in saline soil.

## 5. Conclusions

In the present study, the effects of environmental variances on the bacterial and fungal communities structure in three saline soils were analyzed and quantified. Fungi were more susceptible to salt than the bacteria based on diversity indexes Shannon and Chao1. Therefore, it was suggested to screen halo-tolerant plant growth promoting rhizobacteria from the perspective of soil bacteria in Xinjiang saline land. In addition, PCoA and RDA demonstrated that the soil’s microbial community’s structure exhibited significant differences between different salinized soils. Ca^2+^, AP, EC_e_ and AK, as the dominant environmental factors, correlated with the bacterial community structures (*p* < 0.001). These factors were significantly correlated with the relative abundances of Firmicutes and Bacteroidetes, and this finding could provide guidance for the demand for PGPB as a bioindicator in saline soil.

## Figures and Tables

**Table 1:** Soil physicochemical properties of the three salinization levels.

Physicochemical Factor	S1		S2		S3	
	S1_RS	S1_BS	S2_RS	S2_BS	S3_RS	S3_BS
EC_e_ (ds/m)	0.34 ± 0.01 ^c^	0.36 ± 0.03 ^c^	3.24 ± 0.39 ^b^	3.69 ± 0.11 ^b^	6.13 ± 0.72 ^a^	6.72 ± 0.49 ^a^
pH	7.88 ± 0.07 ^c^	7.99 ± 0.07 ^bc^	8.16 ± 0.04 ^bc^	8.23 ± 0.04 ^b^	8.32 ± 0.23 ^ab^	8.58 ± 0.29 ^a^
clay content (%)	0.55 ± 0.23 ^a^	0.57 ± 0.12 ^a^	0.53 ± 0.19 ^a^	0.67 ± 0.10 ^a^	0.90 ± 0.67 ^a^	0.61 ± 0.03 ^a^
silt content (%)	26.72 ± 2.88 ^a^	25.69 ± 1.57 ^a^	30.91 ± 2.15 ^a^	33.09 ± 1.37 ^a^	32.47 ± 9.25 ^a^	30.95 ± 2.70 ^a^
sand content (%)	72.73 ± 3.10 ^a^	73.74 ± 1.67 ^a^	68.56 ± 2.30 ^a^	66.24 ± 1.47 ^a^	66.63 ± 9.92 ^a^	68.44 ± 2.73 ^a^
K^+^ (mg/kg)	24.00 ± 2.29 ^e^	41.5 ± 2.18 ^d^	63.67 ± 12.75 ^c^	76.50 ± 6.38 ^b^	67.33 ± 5.84 ^bc^	123.83 ± 1.04 ^a^
Na^+^ (mg/kg)	27.00 ± 2.29 ^e^	97.33 ± 3.75 ^e^	271.00 ± 36.17 ^d^	2890.00 ± 193.08 ^b^	652.67 ± 37.44 ^c^	3334.67 ± 63.49 ^a^
Ca^2+^ (mg/kg)	158.20 ± 5.78 ^d^	151.64 ± 2.73 ^d^	495.75 ± 18.43 ^c^	1132.00 ± 131.76 ^b^	2101.50 ± 361.50 ^a^	2193.50 ± 161.47 ^a^
Mg^2+^ (mg/kg)	4.19 ± 0.92 ^d^	5.71 ± 1.82 ^d^	261.00 ± 24.98 ^c^	715.42 ± 65.67 ^b^	271.08 ± 94.11 ^c^	1582.50 ± 68.27 ^a^
SOC (g/kg)	12.61 ± 0.20 ^a^	11.23 ± 0.77 ^b^	6.29 ± 0.46 ^c^	6.20 ± 1.11 ^c^	5.49 ± 0.20 ^c^	5.67 ± 0.11 ^c^
AN (mg/kg)	28.24 ± 2.46 ^a^	30.57 ± 4.20 ^a^	20.54 ± 2.14 ^bc^	22.40 ± 4.28 ^b^	16.34 ± 1.07 ^c^	18.90 ± 2.14 ^bc^
AP (mg/kg)	60.33 ± 3.13 ^a^	62.07 ± 1.46 ^a^	52.00 ± 3.51 ^b^	50.17 ± 2.37 ^b^	25.33 ± 1.92 ^d^	34.70 ± 2.01 ^c^
AK (mg/kg)	242.67 ± 9.71 ^a^	242.00 ± 21.52 ^a^	142.33 ± 7.77 ^c^	205.33 ± 13.50 ^b^	109.67 ± 5.86 ^d^	98.33 ± 2.89 ^d^
ALP (mg/g/d)	3.19 ± 0.20 ^a^	3.49 ± 0.37 ^a^	1.43 ± 0.06 ^b^	0.88 ± 0.18 ^c^	0.69 ± 0.06 ^cd^	0.42 ± 0.02 ^d^
Invertase (mg/g/d)	12.36 ± 1.25 ^a^	10.97 ± 1.01 ^b^	4.31 ± 0.32 ^c^	3.24 ± 0.29 ^c^	1.46 ± 0.04 ^d^	1.07 ± 0.14 ^d^
Urease (mg/g/d)	1.57 ± 0.09 ^a^	1.63 ± 0.02 ^a^	0.87 ± 0.06 ^b^	0.75 ± 0.04 ^c^	0.49 ± 0.02 ^b^	0.42 ± 0.01 ^d^
CAT (mL/g)	1.47 ± 0.12 ^a^	1.10 ± 0.06 ^b^	1.00 ± 0.03 ^bc^	0.94 ± 0.06 ^c^	0.85 ± 0.02 ^cd^	0.80 ± 0.02 ^d^

Note: Values represent means ± SEs of three replicates. Different letters indicate significant differences at the *p* < 0.05 level among the different treatments based on one-way ANOVA. Abbreviations: S1, low salinity; S2, medium salinity; S3, high salinity; S1_RS, low salinity of rhizosphere soil; S1_BS, low salinity of bulk soil; S2_RS, medium salinity of rhizosphere soil; S2_BS, medium salinity of bulk soil; S3_RS, high salinity of rhizosphere soil; S3_BS, high salinity of bulk soil; EC_e_, saturated electrical conductivity; SOC, soil organic carbon; AN, available nitrogen; AP, available phosphorus; AK, available kalium; ALP, alkaline phosphatase; CAT, catalase.

**Table 2:** The relationships between the soil physicochemical properties, the Shannon diversity and Chao1 indexes and the OTU richness of the microbial communities using Spearman’s correlation analysis.

Variable	Shannon		Chao1		OTU richness	
	Bacteria	Fungi	Bacteria	Fungi	Bacteria	Fungi
EC_e_	−0.543 *	−0.652 **	0.609 **	−0.922 **	0.627 **	−0.937 **
pH	−0.616 **	−0.025	−0.293	−0.387	−0.077	−0.372
clay	−0.090	−0.014	0.131	0.029	0.019	0.072
silt	−0.086	−0.441	0.538 *	−0.350	0.408	−0.317
sand	0.049	0.408	−0.499 *	0.300	−0.381	0.268
K^+^	−0.576 *	−0.571 *	0.501 *	−0.765 **	0.515 *	−0.764 **
Na^+^	−0.579 *	−0.591 **	0.606 **	−0.779 **	0.528 *	−0.772 **
Ca^2+^	−0.586 *	−0.668 **	0.568 *	−0.913 **	0.535 *	−0.916 **
Mg^2+^	−0.536 *	−0.513 *	0.662 **	−0.724 **	0.535 *	−0.735 **
SOC	0.687 **	0.709 **	−0.488 *	0.920 **	−0.400	0.926 **
AN	0.537 *	0.694 **	−0.461	0.783 **	−0.464	0.779 **
AP	0.543 *	0.744 **	−0.562 *	0.915 **	−0.527 *	0.936 **
AK	0.597 **	0.539*	−0.647 **	0.890 **	−0.737 **	0.896 **
ALP	0.670 **	0.596 **	−0.558 *	0.891 **	−0.526 *	0.895 **
Invertase	0.719 **	0.659 **	−0.514 *	0.905 **	−0.508 *	0.908 **
Urease	0.660 **	0.652 **	−0.515 *	0.901 **	−0.535 *	0.890 **
CAT	0.643 **	0.676 **	−0.568 *	0.913 **	−0.604 **	0.913 **

Note: * *p* < 0.05; ** *p* < 0.01. Abbreviations: EC_e_, saturated electrical conductivity; SOC, soil organic carbon; AN, available nitrogen; AP, available phosphorus; AK, available kalium; ALP, alkaline phosphatase; CAT, catalase.

## Data Availability

The raw data presented in this study are available on request from the corresponding author. The data are not yet publicly available since the project is still ongoing.

## Supplementary Materials

The following are available online at https://www.mdpi.com/article/10.3390/biology10111114/s1, Table S1: Relative abundances of phyla in Bacteria across all various soil salt gradients. Asterisk indicates sequences classified to the domain Bacteria but not to a specific phylum; Table S2: Relative abundances of phyla in Fungi across all various soil salt gradients; Table S3: Analysis of similarity (ANOSIM); Table S4: Diversity indices of soil microbial communities across the soils; Table S5: Correlations (r) and significance (P) determined by Mantel tests, between the microbial community composition and various soil environmental variables; Figure S1: Rarefaction curves for bacterial and fungal OTUs, clustering at 97% sequence similarity at the three salinization level. (a) Bacteria; (b) Fungi; Figure S2: Rank-abundance curves for bacterial and fungal OTUs in the soil samples taken at the three salinization level. (a) Bacteria; (b) Fungi; Figure S3: (a–d) Linear regression relationships between main environmental factor Ca^2+^, AP, ECe, AK and Shannon of bacterial operational taxonomic units; (e–h) Linear regression relationships between main environmental factor AP, urease, Ca^2+^, ECe and Shannon of fungal operational taxonomic units.
