# Supplementary material for: Responses of the Soil Microbial Community to Salinity Stress in Maize Fields
Source: Biology (Basel). 2021 Oct 29;10(11):1114. doi: 10.3390/biology10111114 (PMC8614889; doi:10.3390/biology10111114)
Supplement: Supplementary file 1 [file biology-10-01114-s001.zip › biology-1417042-supplementary.pdf]

Supplementary Materials

Processing of sequencing data 1

Table S1 Relative abundances of phyla in Bacteria across all various soil salt gradients. Asterisk indicates sequences classified to the domain Bacteria but not to a specific phylum........................3

Table S2 Relative abundances of phyla in Fungi across all various soil salt gradients....................5

Table S3 Analysis of similarity (ANOSIM)..6

Table S4 Diversity indices of soil microbial communities across the soils. 7

Table S5 Correlations (*r*) and significance (*P*) determined by Mantel tests, between the microbial community composition and various soil environmental variables. 8

Figure S1 Rarefaction curves for bacterial and fungal OTUs, clustering at 97% sequence similarity at the three salinization level. a) Bacteria; b) Fungi..10

Figure S2 Rank-abundance curves for bacterial and fungal OTUs in the soil samples taken at the three salinization level. a) Bacteria; b) Fungi. 11

Figure S3 a), b), c) and d) Linear regression relationships between main environmental factor Ca^{2+}, AP, EC_e, AK and Shannon of bacterial operational taxonomic units; e), f), g) and h) Linear regression relationships between main environmental factor AP, urease, Ca^{2+}, EC_e and Shannon of fungal operational taxonomic units...13

Processing of sequencing data

For bacteria, the PCR program was as follows: 1 cycles of 3 min initial denaturation at 95°C; 25 cycles of denaturation at 95°C (30s), annealing at 56°C (30s), elongation at 72°C (45s); and a final extension at 72°C for 10 min, 10°C until halted by user. PCR reactions were performed in 20 μL mixtures containing 4 μL of 5 × Pyrobest Buffer, 2 μL of 2.5 mM dNTPs, 0.8 μL of each primer (5 μM), 0.4 μL of FastPfu Polymerase, 0.2μL of BSA, 10 ng of Template DNA, and added ddH_2O to 20 μL. All samples were amplified in triplicate. Triplicate PCR amplicons were pooled together and detected by electrophoresis in a 2% (w/v) agarose gel. PCR products with bright band between 400 and 450bp were mixed in equal density ratios and purified with TruSeqTM DNA Sample Prep Kit. The amplicon mixture sequenced on gene monitoring platform (MiSeq PE300, Illunima, US) (Majorbio Company in Beijing, China).

For fungi, the PCR program was as follows: The amplification program for PCR consisted of 1 cycles of 3 min initial denaturation at 95°C; 25 cycles of denaturation at 95°C (30s), annealing at 56°C (30s), elongation at 72°C (45s); and a final extension at 72°C for 10 min, 10°C until halted by user. PCR reactions were performed in 20 μL mixtures containing 2 μL of 10 ×Buffer, 2 μL of 2.5 mM dNTPs, 0.8 μL of each primer (5 μM), 0.2 μL of rTaq Polymerase, 0.2μL of BSA, 10 ng of Template DNA, and added ddH_2O to 20 μL. All samples were amplified in triplicate. Triplicate PCR amplicons were pooled together and detected by electrophoresis in a 2% (w/v) agarose gel. Each PCR reaction was sent to the Majorbio Company (Beijing, China).

The extraction of high-quality sequences was first conducted with the QIIME package (Quantitative Insights Into Microbial Ecology) (v1.2.1). Raw sequences were quality checked to

remove low-quality reads, short reads and chimeras. Potential chimera sequences were detected using usearch (version 7.0 http://drive5.com/uparse/). The obtained sequences were further denoised. Sequences of the remaining samples were clustered into operational taxonomic units (OTUs) at 97% of sequence identity. The OTU taxonomy was assigned using Silva (Release132 http://www.arb-silva.de). The abundant and representative OTUs were aligned with GeneBank (NCBI). Sequence analysis was carried out using the microbial diversity analytical software in Majorbio Cloud Platform (https://cloud.majorbio.com/)

Table S1 Relative abundances of phyla in Bacteria across all various soil salt gradients. Asterisk indicates sequences classified to the domain Bacteria but not to a specific phylum.

Phylum	S1		S2		S3	
	S1_RS	S1_BS	S2_RS	S2_BS	S3_RS	S3_BS
Firmicutes	5.01±1.75b	5.57±0.88b	7.79±1.49b	11.56±3.49b	16.15±2.64a	18.53±4.65a
Acidobacteria	14.18±0.58a	12.78±3.55a	8.29±2.54a	7.11±4.12a	3.53±0.66b	2.77±1.47b
Gemmatimonadetes	6.29±0.71a	7.47±2.03a	6.23±0.34a	6.11±0.26a	6.81±0.96a	7.07±0.56a
Bacteroidetes	1.59±0.54b	1.7±0.21b	3.72±1.32b	4.26±1.89b	3.41±0.60a	9.90±6.84a
Planctomycetes	1.52±0.16a	1.49±0.59a	2.21±0.50a	2.17±1.34a	1.15±0.14a	1.43±0.19a
Rokubacteria	1.61±0.31a	1.33±0.09a	1.52±0.46a	1.39±0.34a	0.50±0.13b	0.43±0.36b
Patescibacteria	0.62±0.23b	0.56±0.17b	0.71±0.19b	0.49±0.14b	1.59±0.45a	0.56±0.02b
Verrucomicrobia	0.61±0.38ab	0.53±0.35b	0.58±0.17b	0.28±0.17b	1.17±0.59ab	0.27±0.10b
Nitrospirae	0.90±0.12a	0.68±0.19a	0.85±0.33a	0.50±0.03a	0.15±0.05b	0.19±0.06b
Deinococcus-Thermus	0.07±0.03b	0.17±0.11b	0.19±0.03b	0.36±0.19b	1.30±0.33b	1.84±0.38a
Entotheonellaeota	0.33±0.04b	0.31±0.09b	0.68±0.11a	0.55±0.15a	0.28±0.10b	0.27±0.09b
Bacteria*	0.30±0.03b	0.30±0.03b	0.27±0.13b	0.28±0.17b	0.64±0.26a	0.70±0.16a
Cyanobacteria	0.23±0.05a	0.72±0.68a	0.11±0.03b	0.10±0.04b	0.22±0.11ab	0.18±0.05b
Latescibacteria	0.17±0.05a	0.13±0.02a	0.32±0.16a	0.27±0.19a	0.09±0.10b	0.05±0.04b
Armatimonadetes	0.21±0.10a	0.21±0.06a	0.1±0.02a	0.09±0.02a	0.03±0.01b	0.01±0.01b
Halanaerobiaeota	0.16±0.07a	0.14±0.04a	0.03±0.01b	0.02±0.01b	0.10±0.05a	0.12±0.02a

BRC1	0.05±0.02b	0.06±0.04b	0.08±0.02b	0.10±0.03a	0.14±0.03a	0.10±0.04ab
Dependentiae	0.04±0.05a	0.02±0.01b	0.06±0.02a	0.08±0.08a	0.06±0.01a	0.11±0.03a
Elusimicrobia	0.10±0.02a	0.06±0.02ab	0.06±0.03ab	0.03±0.02b	0.04±0.01b	0.05±0.03b
Chlamydiae	0.02±0.00b	0.03±0.02b	0.05±0.06b	0.04±0.03b	0.05±0.02b	0.12±0.02a
Hydrogenedentes	0.01±0.01c	0.01±0.01c	0.05±0.02a	0.04±0.03b	0.04±0.02b	0.08±0.01a
Tenericutes	0.05±0.01ab	0.06±0.03a	0.02±0.00b	0.03±0.02b	0.03±0.01b	0.05±0.02ab
WS2	0.05±0.03	0.04±0.02	0.03±0.01	0.01±0.02	0.01±0.01	-
Dadabacteria	0.02±0.01a	0.01±0.01a	0.01±0.01a	0.01±0.01a	0.01±0.01a	0.02±0.01a
Fibrobacteres	0.03±0.03a	0.02±0.02a	0.01±0.01b	0.00±0.01b	0.01±0.01b	0.00±0.01b
GAL15	0.02±0.02a	0.01±0.02a	-	-	-	-
WPS-2	0.02±0.01a	0.01±0.01b	0.02±0.01a	-	0.02±0.01a	-
Epsilonbacteraeota	-	-	-	-	0.00±0.01a	-
Margulisbacteria	-	-	-	-	-	0.01±0.01a
Omnitrophicaeota	0.00±0.01a	-	0.01±0.01a	-	-	0.01±0.01
Poribacteria	-	-	-	-	-	-
Spirochaetes	-	-	0.01±0.01a	0.00±0.01a	0.01±0.01a	0.01±0.01a
Zixibacteria	-	0.01±0.01a	-	0.00±0.01a	-	-
Firmicutes	5.01±1.75b	5.57±0.88b	7.79±1.49b	11.56±3.49b	16.15±2.64a	18.53±4.65a
Acidobacteria	14.18±0.58a	12.78±3.55a	8.29±2.54a	7.11±4.12a	3.53±0.66b	2.77±1.47b
Gemmatimonadetes	6.29±0.71a	7.47±2.03a	6.23±0.34a	6.11±0.26a	6.81±0.96a	7.07±0.56a

Note: RS, rhizosphere soil; BS, bulk soil, ab The different letters represent significant differences (*P* < 0.05) in three salinization levels.

Table S2 Relative abundances of phyla in Fungi across all various soil salt gradients.

Phylum	S1		S2		S3	
	S1_RS	S1_BS	S2_RS	S2_BS	S3_RS	S3_BS
Ascomycota	83.42±3.30b	86.41±2.9ab	91.65±0.46ab	96.24±2.19a	82.39±14.18	95.65±3.97a
Mortierellomycota	11.02±2.24a	9.52±4.75a	6.85±1.10ab	2.69±1.69b	0.43±0.26b	1.22±1.68b
Basidiomycota	3.13±0.82a	1.90±2.18ab	0.37±0.23b	0.23±0.06b	1.49±1.40ab	2.64±2.20ab
Chytridiomycota	0.22±0.21a	0.23±0.37a	0.01±0.01a	0.10±0.16a	8.30±14.38a	0.01±0.02a
Glomeromycota	0.06±0.05b	0.02±0.03b	0.01±0.01b	0.01±0.01b	2.88±2.66a	0.03±0.04b
Olpidiomycota	0.02±0.02a	0.05±0.03a	0.09±0.15a	0.01±0.01a	-	-
Blastocladiomycota	-	0.02±0.03a	-	0.01±0.02a	-	-
Cercozoa	0.01±0.01a	-	-	-	0.01±0.01a	0.00±0.01a
Kickxellomycota	0.02±0.02a	-	-	-	-	-

Table S3 Analysis of similarity (ANOSIM).

Group	Bacteria		Fungi	
	R-value	*P*-value	*R*-value	*P*-value
S1/S2	1.000	**0.002**	0.943	**0.003**
S1/S3	1.000	**0.003**	1.000	**0.002**
S2/S3	1.000	**0.005**	0.885	**0.003**
S1_BS/S1_RS	0.111	0.300	0.630	0.100
S1_BS/S2_BS	1.000	0.100	1.000	0.100
S1_BS/S2_RS	1.000	0.100	1.000	0.100
S1_BS/S3_BS	1.000	0.100	1.000	0.100
S1_BS/S3_RS	1.000	0.100	1.000	0.100
S1_RS/S2_BS	1.000	0.100	0.889	0.100
S1_RS/S2_RS	1.000	0.100	1.000	0.100
S1_RS/S3_BS	1.000	0.100	1.000	0.100
S1_RS/S3_RS	1.000	0.100	1.000	0.100
S2_BS/S2_RS	0.481	0.100	0.037	0.500
S2_BS/S3_BS	1.000	0.100	0.519	0.100
S2_BS/S3_RS	1.000	0.100	0.963	0.100
S2_RS/S3_BS	1.000	0.100	0.963	0.100
S2_RS/S3_RS	1.000	0.100	1.000	0.100

Note: S1 including S1_BS and S1_RS; S2 including S2_BS and S2_RS; S3 including S3_BS and S3_RS. Effects of three salinization levels on the bacterial and fungal community structure (OTU level) were calculated using ANOSIM (analysis of similarities) via the vegan package in R (v.3.2.5).

Table S4 Diversity indices of soil microbial communities across the soils.

Classified	Group	Number of OTUs	Shannon	Chao1	coverage
Bacteria	S1_RS	2073±85b	6.52±0.04a	2664.51±108.29b	97.15c
	S2_RS	2384±81a	6.51±0.08a	3099.71±213.76a	97.70b
	S3_RS	2312±111ab	6.07±0.07b	3013.14±160.1a	98.72a
	S1_BS	2043±65b	6.41±0.01ab	2641.48±75.5b	96.94c
	S2_BS	2101±199b	6.29±0.35ab	2968.36±233.84a	96.80c
	S3_BS	2400±123a	5.99±0.09b	3009.57±102.09a	98.66a
Fungi	S1_RS	432±2a	3.89±0.19a	456.00±9.94a	99.94a
	S2_RS	218±5b	3.26±0.29b	238.93±8.28b	99.96a
	S3_RS	130±26c	2.51±0.34c	136.00±26.32c	99.99a
	S1_BS	421±23a	3.78±0.13ab	445.54±25.97a	99.93a
	S2_BS	216±6b	2.93±0.49bc	237.21±18.32b	99.97a
	S3_BS	144±19c	3.36±0.31ab	153.67±19.35c	99.99a

Table S5 Correlations (*r*) and significance (*P*) determined by Mantel tests, between the microbial community composition and various soil environmental variables.

Bacteria			Fungi		
Variable	*r*	*P*	Variable	*r*	*P*
**Ca^{2+}**	**0.906**	**0.001**	**AP**	**0.798**	**0.001**
**AP**	**0.883**	**0.001**	**urease**	**0.773**	**0.001**
**EC_e**	**0.846**	**0.001**	**Ca^{2+}**	**0.772**	**0.001**
**AK**	**0.791**	**0.001**	**ECe**	**0.756**	**0.001**
urease	0.787	0.001	invertase	0.741	0.001
invertase	0.753	0.001	ALP	0.66	0.001
ALP	0.656	0.001	AK	0.642	0.001
SOC	0.547	0.001	SOC	0.629	0.001
Na^{+}	0.539	0.001	AN	0.495	0.001
K^{+}	0.512	0.001	CAT	0.463	0.001
CAT	0.508	0.001	Na^{+}	0.408	0.002
Mg^{2+}	0.496	0.001	K^{+}	0.276	0.005
AN	0.457	0.001	pH	0.242	0.008
pH	0.347	0.003	Mg^{2+}	0.24	0.001
silt	0.071	0.182	silt	0.18	0.036
sand	0.06	0.216	sand	0.165	0.056
clay	-0.069	0.782	clay	-0.004	0.489

Note: EC_e, saturated electrical conductivity; SOC, soil organic carbon; AN, available nitrogen; AP, available phosphorous; AK,

available potassium; ALP, alkaline phosphatase; CAT, catalase.

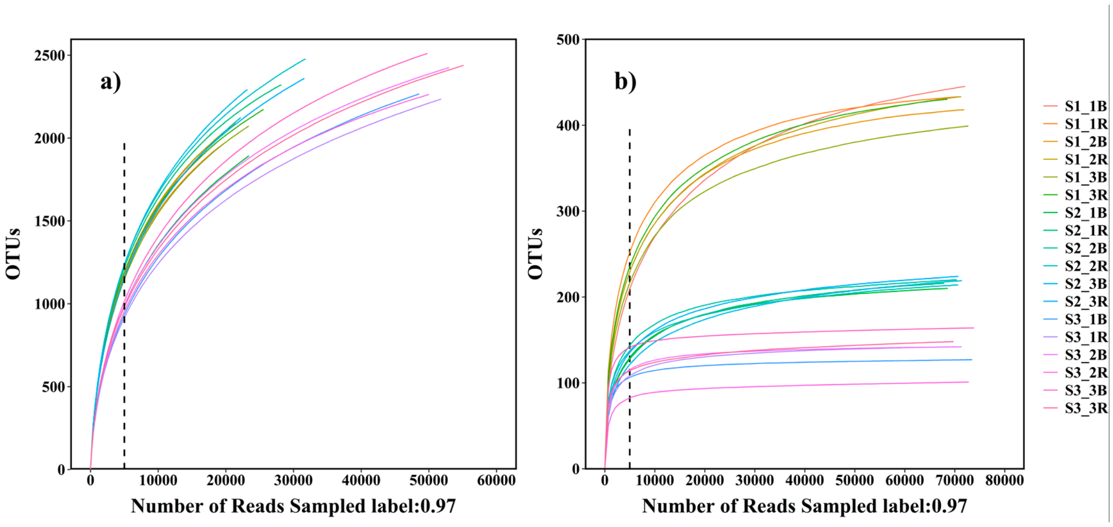

Figure S1 Rarefaction curves for bacterial and fungal OTUs, clustering at 97% sequence similarity at the three salinization level. a) Bacteria; b) Fungi.

Note: S1_1R, the First repetition of the Rhizosphere soil at the S1 sampling point; S1_2R, the Second repetition of the Rhizosphere soil at the S1 sampling point; S1_3R, the Third repetition of the Rhizosphere soil at the S1 sampling point;

S2_1R, the First repetition of the Rhizosphere soil at the S2 sampling point; S2_2R, the Second repetition of the Rhizosphere soil at the S2 sampling point; S2_3R, the Third repetition of the Rhizosphere soil at the S2 sampling point;

S3_1R, the First repetition of the Rhizosphere soil at the S3 sampling point; S3_2R, the Second repetition of the Rhizosphere soil at the S3 sampling point; S3_3R, the Third repetition of the Rhizosphere soil at the S3 sampling point;

S1_1B, the First repetition of the Bulk soil at the S1 sampling point; S1_2B, the Second repetition of the Bulk soil at the S1 sampling point; S1_3B, the Third repetition of the Bulk soil at the S1 sampling point;

S2_1B, the First repetition of the Bulk soil at the S2 sampling point; S2_2B, the Second repetition of the Bulk soil at the S2 sampling point; S2_3B, the Third repetition of the Bulk soil at the S2 sampling point;

S3_1B, the First repetition of the Bulk soil at the S3 sampling point; S3_2B, the Second repetition of the Bulk soil at the S3 sampling point; S3_3B, the Third repetition of the Bulk soil at the S3 sampling point.

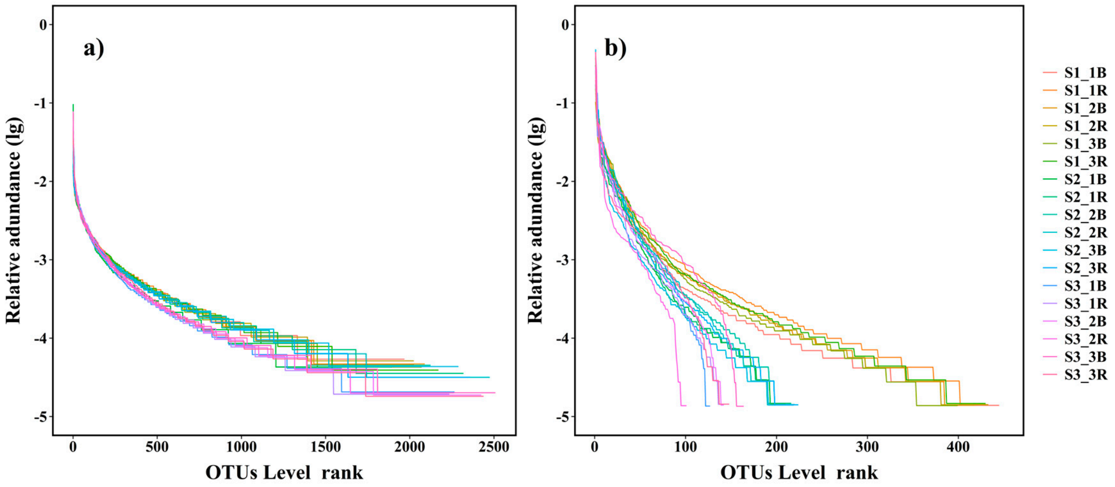

Figure S2 Rank-abundance curves for bacterial and fungal OTUs in the soil samples taken at the three salinization level. a) Bacteria; b) Fungi.

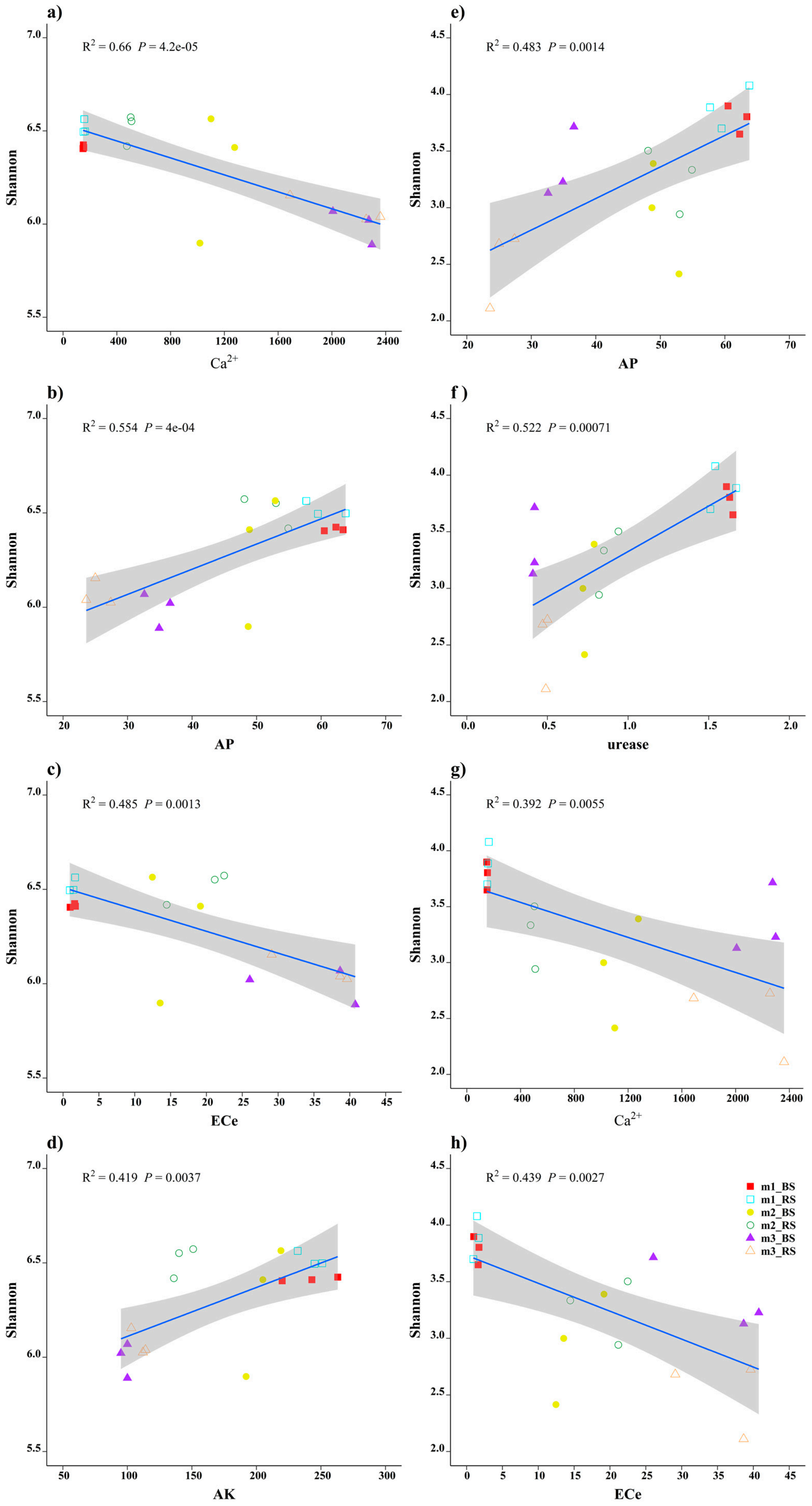

a)
R² = 0.66 P = 4.2e-05
Shannon
Ca2+
e)
R² = 0.483 P = 0.0014
Shannon
AP
b)
R² = 0.554 P = 4e-04
Shannon
AP
f)
R² = 0.522 P = 0.00071
Shannon
urease
c)
R² = 0.485 P = 0.0013
Shannon
ECe
g)
R² = 0.392 P = 0.0055
Shannon
Ca2+
d)
R² = 0.419 P = 0.0037
Shannon
AK
h)
R² = 0.439 P = 0.0027
Shannon
ECe
m1_BS
m1_RS
m2_BS
m2_RS
m3_BS
m3_RS

Figure S3 a), b), c) and d) Linear regression relationships between main environmental factor Ca^{2+}, AP, EC_e, AK and Shannon of bacterial operational taxonomic units; e), f), g) and h) Linear regression relationships between main environmental factor AP, urease, Ca^{2+}, EC_e and Shannon of fungal operational taxonomic units.
